# Rapidly declining trend of signet ring cell cancer of the stomach may parallel the infection rate of *Helicobacter pylori*

**DOI:** 10.1186/s12876-019-1094-x

**Published:** 2019-11-08

**Authors:** Hiroshi Ohyama, Dai Yoshimura, Yosuke Hirotsu, Kenji Amemiya, Hiroyuki Amano, Yuko Miura, Hiroshi Ashizawa, Keiko Nakagomi, Shinya Takaoka, Kenji Hosoda, Yoji Suzuki, Toshio Oyama, Masao Hada, Yuichiro Kojima, Hitoshi Mochizuki, Masao Omata

**Affiliations:** 1 Department of Gastroenterology, Yamanashi Central Hospital, Yamanashi, Japan; 2 Genome Analysis Center, Yamanashi Central Hospital, Yamanashi, Japan; 3 Department of Gastroenterology, Graduate School of Medicine, Chiba University, Chiba, Japan; 4 Department of Pathology, Yamanashi Central Hospital, Yamanashi, Japan; 5 Department of Surgery, Yamanashi Central Hospital, Yamanashi, Japan; 6 University of Tokyo, Tokyo, Japan

**Keywords:** Signet ring cell carcinoma, *Helicobacter pylori*, Genomic detection, Epidemiology, Time series analysis

## Abstract

**Background:**

Studies indicate that gastric cancer (GC) incidence has decreased, whereas signet ring cell carcinoma (SRC) incidence has increased. However, recent trends in GC incidence are unclear. We used our hospital cancer registry to evaluate the changes in the incidence of GC, SRC, and non-SRC (NSRC) over time in comparison to changes in the *H. pylori* infection rates over time.

**Methods:**

We identified 2532 patients with GC enrolled in our registry between January 2007 and December 2018 and statistically analyzed SRC and NSRC incidence. The *H. pylori* infection rate in patients with SRC was determined by serum anti-*H. pylori* antibody testing, urea breath test, biopsy specimen culture, and immunohistochemical analysis (IHC) of gastric tissue. Additionally, genomic detection of *H. pylori* was performed in SRCs by extracting DNA from formalin-fixed paraffin-embedded gastric tissue and targeting 16S ribosomal RNA of *H. pylori.*

**Results:**

Overall, 211 patients had SRC (8.3%). Compared with patients with NSRC, those with SRC were younger (*P* <  0.001) and more likely to be female (*P* <  0.001). Time series analysis using an autoregressive integrated moving average model revealed a significant decrease in SRC (*P* <  0.001) incidence; NSRC incidence showed no decline. There was no difference in *H. pylori* infection prevalence between the SRC and NSRC groups. IHC and genomic methods detected *H. pylori* in 30 of 37 (81.1%) SRCs.

**Conclusions:**

Reduction in *H. pylori* infection prevalence may be associated with the decrease in the incidence of SRC, which was higher than that of NSRC.

## Background

Gastric cancer (GC) is the fifth most frequently diagnosed cancer and the third leading cause of cancer-related death worldwide [1]. Histologically, GC is classified into intestinal and diffuse types [2]. The intestinal type is related to corpus-dominant gastritis with intestinal metaplasia, whereas the diffuse type usually originates from superficial pangastritis without atrophy [3]. The intestinal type is often associated with *Helicobacter pylori* (*H. pylori*) infection, while the diffuse type is more often associated with genetic abnormalities [4].

Signet ring cell (SRC) carcinoma is a form of adenocarcinoma whose histologic diagnosis is based on microscopic characteristics defined by the World Health Organization (WHO) [1]. SRC is classified as diffuse type and non-SRC (NSRC) is mostly classified as intestinal type according to Lauren’s classification [5]. SRC and NSRC are considered distinct biologic entities originating from different pathways of carcinogenesis [5, 6].

Epidemiologically, the worldwide decline in GC incidence has mainly been associated with a decrease in intestinal-type GC in western countries [7]. By contrast, the incidence of diffuse-type GC, particularly SRC, is reported to have increased [7, 8]. SRC represented 28–70% of GC in western countries [8–10]. Moreover, SRC incidence has significantly increased by 6.5% per year on average, representing an approximately 10-fold increase from 1973 to 2000 [8]. However, the trends in the incidence of SRC after 2000 have not been fully examined.

GC rates in Japan are one of the highest in the world, presumably because of the high *H. pylori* infection rate [11]. However, the prevalence of *H. pylori* infection has drastically decreased, i.e., from 80 to 90% in older generations born before around 1950 to < 10% currently in individuals aged < 20 years [12]. According to a previous study using Joinpoint regression analysis, the prevalence of *H. pylori* infection in subjects born between 1927 and 1949 decreased from 48.6 to 43.5%, with a decline of 0.2% per year. Subsequently, a rapid decline in the prevalence of *H. pylori* infection in those born between 1949 (43.5%) and 1961 (22.7%) was found, with a decline of 1.7% per year. Another decrease was observed between 1961 (22.7%) and 1988 (6.3%), with a decline of 0.6% per year. The drastic decline in the prevalence of *H. pylori* infection by birth year can be explained by the change in sanitary conditions during childhood, when *H. pylori* infection is predominantly acquired [13].

Treatment for *H. pylori* infection was developed in 2000; however, governmental health insurance plan coverage of this treatment was limited to patients with peptic ulcers. In February 2013, the indications for treatment were expanded to include chronic gastritis. Subsequently, it was estimated that the number of patients with successful *H. pylori* eradication drastically increased after 2013, doubling to > 1,300,000 from approximately 650,000 per year between 2001 and 2012 [14].

In 2006, the Japanese government began to encourage hospitals to create cancer registries. In 2013 the government changed the law to mandate these registries, and enforcement began in 2016. Our hospital voluntarily initiated a cancer registry in 2006, and since then we have accumulated information on all malignant neoplasms seen at our hospital. From 2007 to 2018, our registry enrolled 22,674 patients with various cancer types. Using the registry data, we evaluated the changes in the incidence of GC, SRC, and NSRC over time in comparison to changes in *H. pylori* infection rates over time using time series analysis.

## Methods

### Background

Our hospital covers the central part of Kofu City (population 400,000) in the highland area of Mt. Fuji, which is 100 km west of Tokyo. Compared with the people in the metropolitan area of Tokyo, the people in our area tend to stay here for life, making it easier to obtain follow-up studies on the patients. Approval for this retrospective review study was obtained from the Institutional Review Board at Yamanashi Central Hospital. The requirement for written informed consent was waived by the institutional review board. Since clinical data and previously collected samples were used in this retrospective study, the use of an opt-out consent method was approved by the institutional review board.

### Pathological confirmation of SRC and NSRC

Starting in January 2007, we registered all patients with histologically proven GC. By the end of 2018, 2532 patients had been enrolled, and all of them underwent surgery, endoscopic submucosal dissection, and/or biopsy for diagnosis and treatment. All SRC and NSRC cases were confirmed by pathological diagnosis. SRC was described according to the WHO classification, i.e., poorly cohesive tumor cells with prominent cytoplasmic mucin and a crescent-shaped eccentrically placed nucleus [15].

### Clinical features

Age, sex, and clinical data (including dates of diagnosis, histopathological diagnosis, the Union for International Cancer Control TNM classification, types of treatment, and dates of death) of patients were all documented in the databases of the in-hospital cancer registry. With the help of the Japanese government and the recently established law, we obtained the mortality data of all 2532 patients. Background gastric mucosal atrophy was classified into four grades of severity (i.e., normal, mild, moderate, and marked) on the basis of the updated Sydney system by an expert pathologist (T. O.) using formalin-fixed and paraffin-embedded (FFPE) tissue [16].

### Detection of *H. pylori* infection

#### Serological testing

Blood samples of patients with GC were collected and used for serology testing; anti-*H. pylori* antibodies were detected by latex agglutination turbidimetry (BML, Tokyo, Japan). Patients with an anti-*H. pylori* antibody titer > 10 U/mL were classified as *H. pylori* antibody-positive.

#### Urea breath test (UBT).

The patients ingested 13C-labeled urea (100 mg), which was converted to ^13^CO_2_ by the urease enzyme produced by *H. pylori* if the bacteria were present in the stomach (BML). The released ^13^CO_2_ diffused into the blood and was released from the lungs. The expired air was collected 20 min after ^13^C-labeled urea ingestion to measure the ^13^C/^12^C ratio. A positive result of the UBT was defined as a difference between baseline and test samples of > 2.5‰.

#### Bacterial culture

Gastric mucosa specimens were collected from patients by endoscopic biopsy. The processed specimens were smeared in *Helicobacter* agar media (Nissui Pharmaceutical, Tokyo, Japan) and horse blood agar media (Kyokuto Pharmaceutical Industrial, Tokyo, Japan) and cultivated at 37 °C in a microaerophilic environment.

#### Immunohistochemical detection

Immunohistochemical (IHC) detection was performed using 3-μm-thick serial sections of FFPE tissue from resections or biopsies. The sections were deparaffinized, and antigen activation was performed by heat treatment in ethylenediaminetetraacetic acid solution at pH 8.0. Protein expression was evaluated on the 3-μm-thick FFPE sections with rabbit polyclonal anti-*H. pylori* antibodies (diluted 1:10; Institute of Immunology, Tokyo, Japan) using the Ventana BenchMark ULTRA (Roche, Tucson, Arizona) [17, 18].

#### Genomic detection of *H. pylori* 16S ribosomal RNA (rRNA)

For detection of *H. pylori*, we obtained FFPE tissues from patients with SRC. DNA was extracted from 10-μm-thick sections of FFPE using FormaPure DNA (Beckman Coulter, USA) with magnetic beads according to the manufacturer’s instructions. DNA concentration was determined using the Nano Drop 2000 spectrophotometer (Thermo Fisher Scientific, Waltham, MA). Primers were designed to amplify the *H. pylori* 16S rRNA region and generate 110 bp polymerase chain reaction (PCR) products. Primer sequences were: forward (5′-.CTGGAGAGACTAAGCCCTCC-3′); reverse (5′-ATTACTGACGCTGATTGTGC-3′) [19]. Each PCR was performed with 50 μL of reaction solution containing 45 μL of Platinum™ PCR SuperMix High Fidelity (Thermo Fisher Scientific), 1 μL each of forward and reverse primers, 1 μL of nuclease-free water, and 2 μL of extracted DNA. Reactions were subject to thermal cycling with the following conditions: 94 °C for 2 min, 45 cycles of 94 °C for 15 s, 60 °C for 15 s, and 68 °C for 30 s. To determine the *H. pylori-*specific amplicon, 5 μL of the PCR products was separated by electrophoresis on 2% agarose gel, stained with ethidium bromide and visualized on an FAS IV UV Illuminator (NIPPON Genetics, Japan) [20, 21]. Moreover, direct-sequencing was performed by the Sanger method using 16S rRNA PCR products [22]. PCR products were purified using AMPure XP (Beckman Coulter) according to the manufacturer’s instructions. Sanger sequencing was performed with BigDye Terminator v3.1 Cycle Sequencing Kit (Thermo Fisher Scientific) using forward or reverse primers. PCR products were purified BigDye XTerminator™ Purification Kit and subsequently analyzed by 3500 Genetic Analyzer (Thermo Fisher Scientific). The nucleotide sequences were aligned by BLAST (https://blast.ncbi.nlm.nih.gov/Blast.cgi).

### Statistical analysis

Student’s *t*-test and the Mann–Whitney *U* test were used to compare continuous variables, whereas the chi-squared test and Fisher’s exact test were used for comparison of categorical variables.

To evaluate time series data, namely, the incidence of SRC and NSRC, time series analysis was performed using the autoregressive integrated and moving average (ARIMA) model [23]. The ARIMA model-building process was designed to take advantage of associations in the sequentially lagged relationships that usually exist in data collected periodically. When stationarity was achieved using differencing, the ARIMA model was built, and the fitted ARIMA model was used to forecast the incidence.

A probability (*P)* value of < 0.05 was considered statistically significant. All statistical analyses were performed using SPSS software version 20.0 (IBM-SPSS, Inc., Chicago, IL, USA) and R software packages (version 3.3.3; R Development Core Team).

## Results

### Clinical features of SRC and NSRC

Histopathological analysis of the 2532 patients with GC prospectively registered from January 2007 to December 2018 revealed 211 cases of SRC (8.3%) and 2321 of NSRC (91.7%). The median age of patients with SRC was 64 years (range 29–94 years) and that of patients with NSRC was 73 years (range 27–99 years) (Table 1). Patients with SRC were significantly younger than those with NSRC (*P* <  0.001). A greater number of female patients were observed among patients with SRC (*P* <  0.001).

**Table 1:** Characteristics of SRC and NSRC patients

Variable	SRC (*n* = 211)	NSRC (*n* = 2321)	*P*
Age median, years (range)	64 (29–94)	73 (27–99)	< 0.001
Gender, n (%)			< 0.001
Female Male	110 (52.1) 101 (47.9)	666 (28.7) 1655 (71.3)	
Clinical Stage, n (%)			0.124*
IA IB IIA IIB IIIA IIIB IIIC IV Unknown	107 (50.7) 33 (15.6) 10 (4.7) 5 (2.4) 8 (3.8) 3 (1.4) 1 (0.5) 29 (13.7) 15 (7.1)	1168 (50.3) 186 (8.0) 124 (5.3) 57 (2.5) 91 (3.9) 34 (1.5) 12 (0.5) 324 (14.0) 325 (14.0)	

*Unknown cases were excluded from the statistical analysis

NSRC, non-signet ring cell carcinoma; SRC, signet ring cell carcinoma

**Fig. 1:**
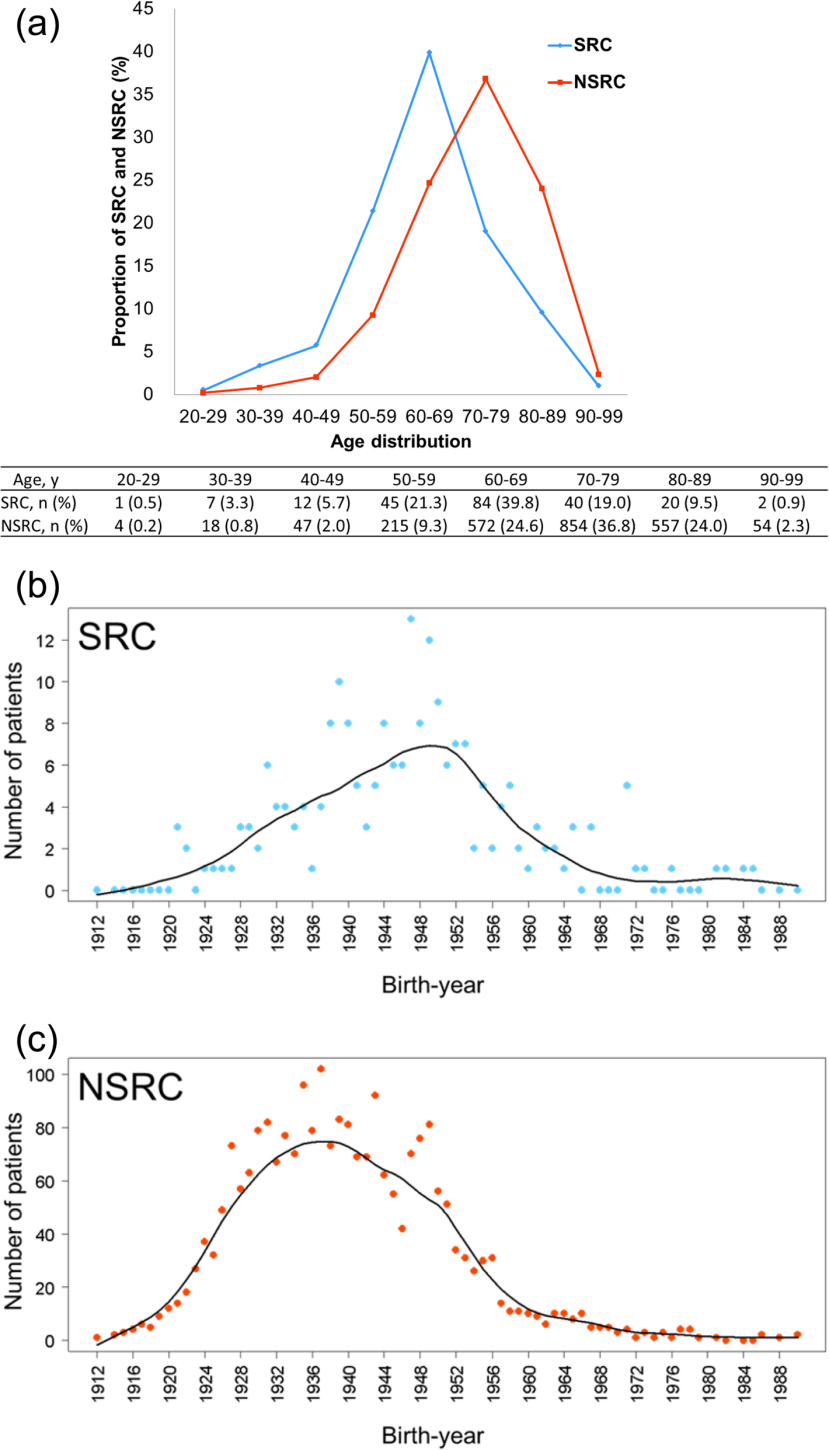
(**a**) Age distribution of patients with SRC (blue line) and NSRC (red line). (**b**) Relationship between birth-year and the number of patients with SRC. (**c**) Relationship between birth-year and the number of patients with NSRC. Curved lines indicate approximate curves

Peak ages associated with SRC and NSRC occurrence were the 60s and 70s, respectively (Fig. 1a). When SRC and NSRC were plotted on the basis of birth year, the peak of SRC was between 1948 and 1952, whereas that of NSRC was between 1936 and 1940 (Fig. 1b and c).

### Annual incidence of SRC and NSRC

**Fig. 2:**
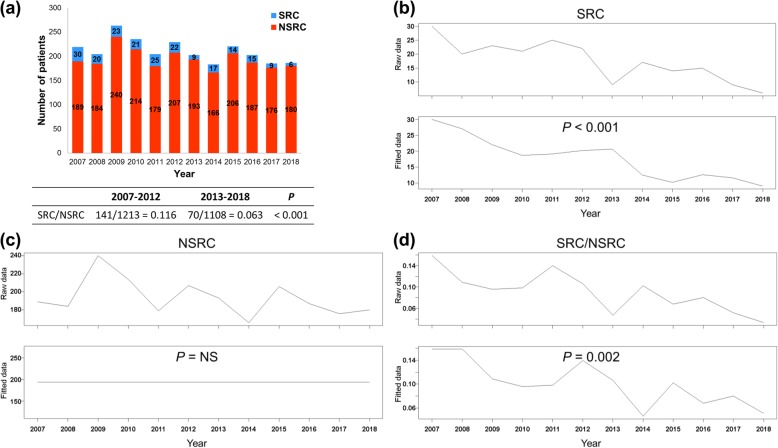
(**a**) The number of registered SRC and NSRC cases from 2007 to 2018 (the ratio of the total number of SRC and NSRC cases during the period of 2007–2012 and 2013–2018). *P* value was calculated by the Mann–Whitney *U* test (*P* < 0.001). **b–d** Time series analysis of the number of SRC (**b**) and NSRC (**c**) cases and the SRC/NSRC ratio (**d**) using autoregressive integrated and moving average (ARIMA) model. Raw data of time series achieved stationarity using differencing. Subsequently, autoregressive moving average model was applied to the series. The fitted model was used to forecast the incidence. The annual number of SRC cases and the SRC/NSRC ratio significantly decreased (*P* < 0.001 and *P* = 0.002, respectively). NS, not significant

Before 2012, the annual number of registered cases of SRC was 20–30, whereas after 2013, the number decreased to 9–17 (Fig. 2a). The annual numbers of registered cases of NSRC were 179 to 240 before 2012 and 176 to 206 after 2013. A comparison of the SRC/NSRC ratio in 2007–2012 with that in 2013–2018 showed that the latter was significantly lower (141/1213 (11.6%) vs. 70/1108 (6.3%), *P* <  0.001 by the Mann–Whitney *U* test) (Fig. 2a).

### Time fluctuation of the incidence of SRC and NSRC

We used the ARIMA model to validate the declining trend of the incidence of SRC by modeling the number of SRC and NSRC using time series data from 2007 to 2018. The annual number of SRCs and the SRC/NSRC ratio significantly decreased (*P* < 0.001 and *P* = 0.002, respectively) (Fig. 2b-d). By contrast, the annual number of NSRCs did not change significantly.

### Clinical detection of *H. pylori* infection

First, we retrospectively investigated the *H. pylori* infection rate using clinical records. Clinically employed methods included anti-*H. pylori* antibody testing, UBT, and bacterial culture. Of 211 SRC cases, only 25 (11.8%) were tested by at least one of the three measures, whereas 729 of 2321 NSRC (31.4%) cases were tested (Table 2). The difference was statistically significant (*P* < 0.001). Interestingly, although small in number, 15 of 25 SRC cases (60.0%) and 387 of 729 NSRC cases (53.1%) tested positive for *H. pylori* with at least one of the three testing methods (Tables 2 and 3). These data indicated that SRC had been regarded as being unrelated to *H. pylori* infection [24, 25]; thus, evaluation for *H. pylori* infection in patients with SRC is less frequent than that in patients with NSRC in the clinical setting. However, compared to that in NSRC, using the three aforementioned measures may yield similar or even higher *H. pylori* infection rates in SRC. Hence, IHC and genomic methods were additionally employed to detect *H. pylori* infection in SRC using archived FFPE gastric tissue in cases in which we could not obtain serum samples, breath, or fresh gastric tissue. We were able to retrieve 37 FFPE samples from 211 patients with SRC (31 surgical specimens and 6 biopsies). Both genomic and IHC analyses of the 37 SRC cases revealed 22 *H. pylori*-positive cases (59.5%) (Table 4). There were 26 cases (70.3%) in which either genomic or IHC analysis was *H. pylori*-positive. Sanger sequencing using 16S rRNA PCR products revealed that sequences of PCR products were matched to *H. pylori* genomic sequence (Additional file 1). Eventually, combining the clinical tests (antibody, UBT, and culture) and the two additional methods (IHC and genomic methods) showed that at least one approach tested positive for *H. pylori* infection in 30 of the 37 SRC cases (81.1%) (Table 4).

**Table 2:** The number of patients with SRC and NSRC who were tested for the presence of *H. pylori* infection

	Subjects in SRC (*n* = 211)	Subjects in NSRC (*n* = 2321)	*P*
Testing for *H. pylori* infection, n (%)			
Ab UBT Bacterial culture At least one	6/211 (2.8) 14/211 (6.6) 11/211 (5.2) 25/211 (11.8)	117/2321 (5.0) 545/2321 (23.5) 486/2321 (20.9) 729/2321 (31.4)	0.155 < 0.001 < 0.001 < 0.001

Ab, anti-*H. pylori* antibody; NSRC, non-signet ring cell carcinoma; SRC, signet ring cell carcinoma; UBT, urea breath test

**Table 3:** Prevalence of *H. pylori* infection in patients with SRC and NSRC who were tested for the presence of *H. pylori* infection

	Positivity in SRC	Positivity in NSRC	*P*
Testing for *H. pylori* infection, n (%)			
Ab UBT Bacterial culture At least one	4/6 (66.7) 5/14 (35.7) 7/11 (63.6) 15/25 (60.0)	47/117 (40.2) 221/545 (40.6) 172/486 (35.4) 387/729 (53.1)	0.231 0.716 0.063 0.496

Ab, anti-*H. pylori* antibody; NSRC, non-signet ring cell carcinoma; SRC, signet ring cell carcinoma; UBT, urea breath test

**Table 4:** Prevalence of *H. pylori* infection in patients with SRC (*n* = 37)

No.	Age range*	Gender*	IHC	Genomic detection	Ab	UBT	Culture	At least one positive
1	50–59	1	+	+	NE	+	+	+
2	50–59	2	+	+	NE	+	NE	+
3	60–69	1	+	+	NE	+	NE	+
4	40–49	2	+	+	+	–	NE	+
5	60–69	1	+	+	+	NE	NE	+
6	60–69	2	+	–	NE	NE	+	+
7	60–69	1	+	+	NE	NE	+	+
8	70–79	1	+	–	NE	NE	+	+
9	70–79	2	+	+	NE	–	NE	+
10	50–59	2	+	+	NE	NE	–	+
11	80–89	2	+	+	NE	NE	–	+
12	30–39	1	+	+	NE	NE	NE	+
13	40–49	1	+	+	NE	NE	NE	+
14	30–39	2	+	+	NE	NE	NE	+
15	40–49	1	+	+	NE	NE	NE	+
16	40–49	1	+	+	NE	NE	NE	+
17	40–49	2	+	+	NE	NE	NE	+
18	30–39	1	+	+	NE	NE	NE	+
19	30–39	1	+	+	NE	NE	NE	+
20	40–49	1	+	+	NE	NE	NE	+
21	40–49	2	+	–	NE	NE	NE	+
22	30–39	2	+	–	NE	NE	NE	+
23	60–69	2	–	+	–	–	NE	+
24	40–49	2	–	+	NE	–	NE	+
25	50–59	2	–	+	NE	–	NE	+
26	50–59	2	–	+	–	NE	NE	+
27	80–89	1	–	–	+	–	–	+
28	60–69	2	–	–	NE	+	NE	+
29	70–79	2	–	–	NE	+	NE	+
30	80–89	1	–	–	NE	NE	+	+
31	70–79	2	–	–	NE	–	NE	–
32	50–59	1	–	–	NE	NE	–	–
33	40–49	2	–	–	NE	NE	NE	–
34	40–49	1	–	–	NE	NE	NE	–
35	30–39	2	–	–	NE	NE	NE	–
36	40–49	1	–	–	NE	NE	NE	–
37	20–29	1	–	–	NE	NE	NE	–
			22/37 (59.5%)	22/37 (59.5%)	3/5 (60.0%)	5/12 (41.7%)	5/9 (55.6%)	30/37 (81.1%)

*Age range was used instead of the exact age and genders were presented by 1 or 2 instead F or M for securing the patients’ anonymity

Ab, anti-*H. pylori* antibody; IHC; immunohistochemical detection; NE, not examined; SRC, signet ring cell carcinoma; UBT, urea breath test

### Background gastric mucosal atrophy in SRC and NSRC

To investigate the background gastric mucosal atrophy in GC, we searched for histopathological findings on resected or biopsied specimens. We obtained 849 specimens, of which 807 were resected specimens (95.1%). Of the 849 specimens, 123 belong to SRC cases and 726 to NSRC cases. Background gastric mucosal atrophy was noted in 109 SRC cases (88.6%) and in 666 NSRC cases (91.7%). No statistical difference in the incidence of background gastric mucosal atrophy between patients with SRC and NSRC was found (*P* = 0.257). However, glandular atrophy was less severe in patients with SRC than in patients with NSRC (*P* < 0.001; Table 5). Remarkably, marked atrophy was observed in only 5.7% (7/123) of SRC and 20.5% (149/726) of NSRC (*P* < 0.001) patients. When the patients with SRC and NSRC were stratified according to glandular atrophy severity, patients with SRC were significantly younger than those with NSRC, except for those in the marked atrophy group (Table 6).

**Table 5:** Histopathological severity of background gastric mucosal atrophy in SRC and NSRC

	SRC (*n* = 123) n (%)	NSRC (*n* = 726) n (%)	*P*
Atrophy			
Normal Mild Moderate Marked	14 (11.4) 62 (50.4) 40 (32.5) 7 (5.7)	60 (8.3) 253 (34.8) 264 (36.4) 149 (20.5)	0.257 0.001 0.411 < 0.001

NSRC, non-signet ring cell carcinoma; SRC, signet ring cell carcinoma

**Table 6:** Age of patients with SRC and NSRC and the histopathological severity of gastric mucosal atrophy

	SRC (*n* = 123) Age mean ± SD (n)	NSRC (*n* = 726) Age mean ± SD (n)	*P*
Atrophy			
Normal Mild Moderate Marked	56.6 ± 13.4 (14) 63.5 ± 11.5 (62) 66.9 ± 12.6 (40) 67.6 ± 12.6 (7)	66.0 ± 12.1 (60) 67.7 ± 11.4 (253) 72.1 ± 9.4 (264) 73.3 ± 9.2 (149)	0.012 0.011 0.001 0.253

NSRC, non-signet ring cell carcinoma; SRC, signet ring cell carcinoma

## Discussion

The morbidity and mortality of patients with GC have drastically decreased in the past 70 years [26]. Up to the 1980s, environmental factors, such as nutrition and socioeconomic conditions, were presumed to play a major role in the development of GC [27]. However, after the discovery of *H. pylori* in 1984 [28], the opinion on carcinogenesis of the stomach completely changed. In 1994, the International Agency for Research on Cancer classified *H. pylori* as a type I (definite) carcinogen in human beings [29]. Although GC is caused by multiple factors, *H. pylori* infection has been regarded as a main risk factor. *H. pylori* has been linked to chronic atrophic gastritis, which is an established precursor of the intestinal type of gastric carcinoma [30]. Thus, elimination of *H. pylori* has been considered the most important goal of GC reduction worldwide [30, 31].

In Japan, a dramatic decline in the prevalence of *H. pylori* infection, possibly attributable to improved hygiene, was observed in those born between 1949 and 1961 (43.5 and 22.7%, respectively) [13]. In addition, the number of patients who have been cured of *H. pylori* infection by drug treatment is rapidly increasing in Japan. The nationwide estimated number of patients who were successfully treated was approximately 0.6 million per year between 2001 and 2012, which rapidly increased to around 1.4 million in 2013 [32].

SRC is a unique type of GC. Our data showed that 211 of 2532 GC cases (8.3%) had SRC. The etiology and pathogenesis of SRC are completely unknown. SRC that is detected late is associated with an extremely poor outcome [7]. Clinically, we often treat relatively young female patients with diffuse-type GC (stage IV).

Several clinical studies have demonstrated the association of *H. pylori* infection with SRC. Asaka et al. reported that 86.4% of diffuse-type GC had a positive serology for *H. pylori* [33], and Kikuchi et al. reported that the frequency of positive *H. pylori* antibody in diffuse-type GC was 87.8% [34]. Huang et al. reviewed ten published studies and reported that 82.2% of patients with diffuse-type GC were seropositive for *H. pylori* [35]. On the contrary, there has been a long-held opinion that SRC is so unique that it is not related to *H. pylori* infection. Buruk et al. reported that *H. pylori* was found in 88% of the intestinal type and in only 55% of the diffuse type (*P* < 0.05) GCs [1, 24, 25]. Moreover, the implementation rate of clinical examination for *H. pylori* infection in SRC was low in this study, reflecting the prevalent opinion that diffuse-type GC or SRC is related to genetic or unknown factors rather than to obvious *H. pylori* infection (Table 2). However, with the addition of genomic detection targeting 16S rRNA of *H. pylori* and IHC detection of *H. pylori* utilizing FFPE, *H. pylori* infection was found in 81.1% of our registered SRC cases (Table 4).

GC is considered the end result of a long-lasting *H. pylori* infection leading to stomach epithelium atrophy with accumulated somatic mutations [36]. Thus, changes in overall GC incidence due to eradication of the bacteria would be expected to occur extremely slowly. Nevertheless, an interesting finding was reported in an institution in Taiwan. A universal hepatitis B vaccination program in 1984 resulted in an immediate 50% decrease in the incidence of hepatocellular carcinoma in children within 13 years [37]. Previously it was thought that such a reduction in hepatocellular carcinoma incidence would take 30 to 40 years. The most common age range for the onset of hepatitis B-related hepatocellular carcinoma was 50 to 60 years, following development of chronic hepatitis and cirrhosis. Thus, the immediate effect was an unexpected result and led to postulating that hepatitis B virus may directly induce hepatocellular carcinoma without preceding chronic hepatitis and cirrhosis.

Of further interest, marked atrophy was less common (5.7%, 7/123) in patients with SRC than in those with NSRC (20.5%, 149/726) (Table 5). These data indicated that despite the rate of *H. pylori* infection being the same as that of NSRC incidence the bacteria may not cause severe atrophy and may trigger SRC in the stomach in the short term instead (Table 6). Several clinical as well as basic studies on the pathogenesis of SRC have been conducted. Uemura et al. reported that diffuse-type GC is associated with active inflammation and not necessarily with marked atrophy [38]. Moreover, several important genetic abnormalities in GC have been reported. Approximately 1–3% of GC occurs because of an inherited gastric cancer predisposition [39]. Linkage analysis has implicated *CDH1* (also known as E-cadherin) mutations in approximately 25% of families with an autosomal-dominant predisposition to diffuse-type GC [40]. The causal role of *H. pylori* infection for specific alterations in DNA methylation patterns was demonstrated in the gastric mucosa of *H. pylori*-infected patients and in GC cell lines [41]. Interestingly, *CDH1* gene methylation is reported in sporadic diffuse-type GC associated with *H. pylori* infection, and the methylation of the *CDH1* promoter could be reversed by the eradication of *H. pylori* [42]. Hence, *H. pylori* eradication therapy may decrease the incidence of diffuse-type GC by suppressing inflammation [43] and specific alterations of DNA methylation.

Our study has several limitations. First, we evaluated patients retrospectively; thus, we could not exclude various biases entirely. Second, this was a single-institutional study, so a small number of patients with SRC were evaluated. However, our data showed that the SRC incidence significantly decreased. Currently, although all available data indicate that the incidence of SRC continues to increase worldwide, no obvious explanation for the direct contribution of *H. pylori* infection to the development of SRC exists. However, studies on the increase in SRC incidence were published before 2005 [7, 8]. The government mandated national registry discussed earlier, by collecting cases from the entire nation of Japan, will enable epidemiological studies which may answer the question of whether SRC incidence is truly decreasing.

## Conclusions

Reduction in SRC incidence seems more rapid than that in overall GC incidence on the basis of the findings of this study. The drastic decline in the prevalence of *H. pylori* infection by the improved hygiene may have contributed to the reduction in SRC. Moreover, eradication of *H. pylori* may hasten the decrease in the incidence of SRC.

## Supplementary information

**Additional file 1. **Representative direct-sequencing result by the Sanger method using 16S rRNA PCR products. Sanger sequencing was performed to determine the nucleotide sequence of 16S rRNA PCR products and the sequences were validated by BLAST (https://blast.ncbi.nlm.nih.gov/Blast.cgi). As a result, sequences of PCR products were matched to *H. pylori* genomic sequence. (TIF 133 kb)

## Data Availability

All data supporting the conclusions of this article are included within the article. The datasets analyzed are available from the corresponding author on reasonable request.

## Abbreviations

ARIMA: the autoregressive integrated and moving average;
FFPE: formalin-fixed and paraffin-embedded
GC: gastric cancer
IHC: immunohistochemical
NSRC: non-signet ring cell carcinoma
PCR: polymerase chain reaction
rRNA: Ribosomal RNA
SRC: signet ring cell carcinoma
UBT: urea breath test
WHO: the World Health Organization
