# Supplementary figures and images for: Rapidly declining trend of signet ring cell cancer of the stomach may parallel the infection rate of Helicobacter pylori
Source: BMC Gastroenterol. 2019 Nov 8;19:178. doi: 10.1186/s12876-019-1094-x (PMC6842265; doi:10.1186/s12876-019-1094-x)

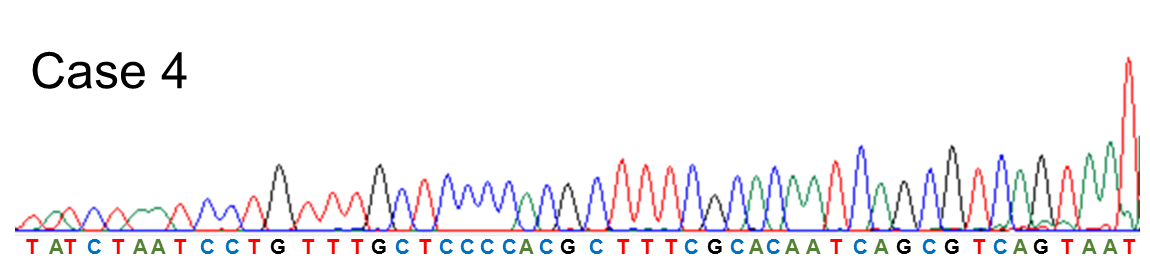

Supplement: Supplementary file 1 — Additional file 1. Representative direct-sequencing result by the Sanger method using 16S rRNA PCR products. Sanger sequencing was performed to determine the nucleotide sequence of 16S rRNA PCR products and the sequences were validated by BLAST (https://blast.ncbi.nlm.nih.gov/Blast.cgi). As a result, sequences of PCR products were matched to H. pylori genomic sequence. (TIF 133 kb) [file 12876_2019_1094_MOESM1_ESM.tif]
